# Working towards sustainable eye care at district hospitals in Viet Nam

**Published:** 2013

**Authors:** Huynh Tan Phuc, Le Quang Tram Tinh

**Affiliations:** Country Manager: The Fred Hollows Foundation, Da Nang city, Viet Nam. phuynh@hollows.org; Senior Project Offcer, The Fred Hollows Foundation, Da Nang city, Viet Nam. **tle@hollows.org**

**Figure:**
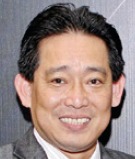
Huynh Tan Phuc

**Figure:**
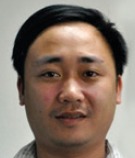
Le Quang Tram Tinh

The shortage of adequate facilities and qualified staff at district and community level means that tertiary eye care services in Viet Nam have been overburdened by high volumes of patients, not all of whom needed tertiary-level care. In Quang Nam province, an additional challenge is the mountainous terrain, which makes travel very difficult.

To help reduce the pressure on tertiary centres and increase access to eye care, the Fred Hollows Foundation (FHF) has been working since 2005 to implement a sustainable model of community eye care (CEC) based at two district hospitals: one in Que Son district (population >80,000) and one in Dai Loc district (population >145,000). This work is supported by Standard Chartered Bank's ‘Seeing is Believing’ Initiative.

## Vision centres

Previously, the eye departments at the two district hospitals mainly provided eye examinations, referrals, and some medical treatments. The CEC project has involved transforming the eye departments into vision centres with several functions:

- coordination of eye health promotion and community screening
- eye examinations and referral to tertiary centres if needed
- treatment of eye disease (including cataract surgery) at the district hospital
- refraction and dispensing of spectacles.

## Reducing the cost of surgery

Strategic procurement procedures, including competitive tender processes, ensured that high quality consumables for surgery could be obtained at reasonable cost. Low-cost, high quality intraocular lenses (IOLs) were imported from FHF-established laboratories in the region, and used for all cataract operations. These IOLs cost only US $5.00, reducing the total cost of a cataract operation to just US $45.00 per eye.

## Quality and efficiency

Local eye health personnel, including cataract surgeons, must undergo on-going training and skills development in order for the project to be sustainable. To achieve this, experienced surgeons were invited to lead a surgical training programme at the hospital, which proved to be very effective. This programme, together with other opportunities for continued professional development (e.g. workshops, refresher training, and mentoring with experienced surgeons), has allowed personnel to network and share knowledge and information. This has helped to improve the outcomes of cataract surgery and ensure that quality and efficiency remain high.

**Figure:**
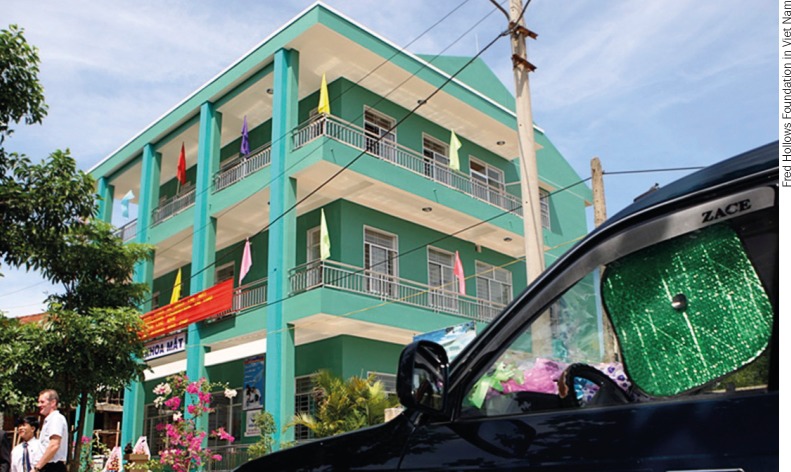
Dai Loc Eye Clinic

It is important to form close partnerships with suppliers and to train staff in the use and maintenance of instruments and equipment. For the hospital to run smoothly and offer high quality services, there has to be adequate supplies and well-maintained equipment and instruments.

**Figure:**
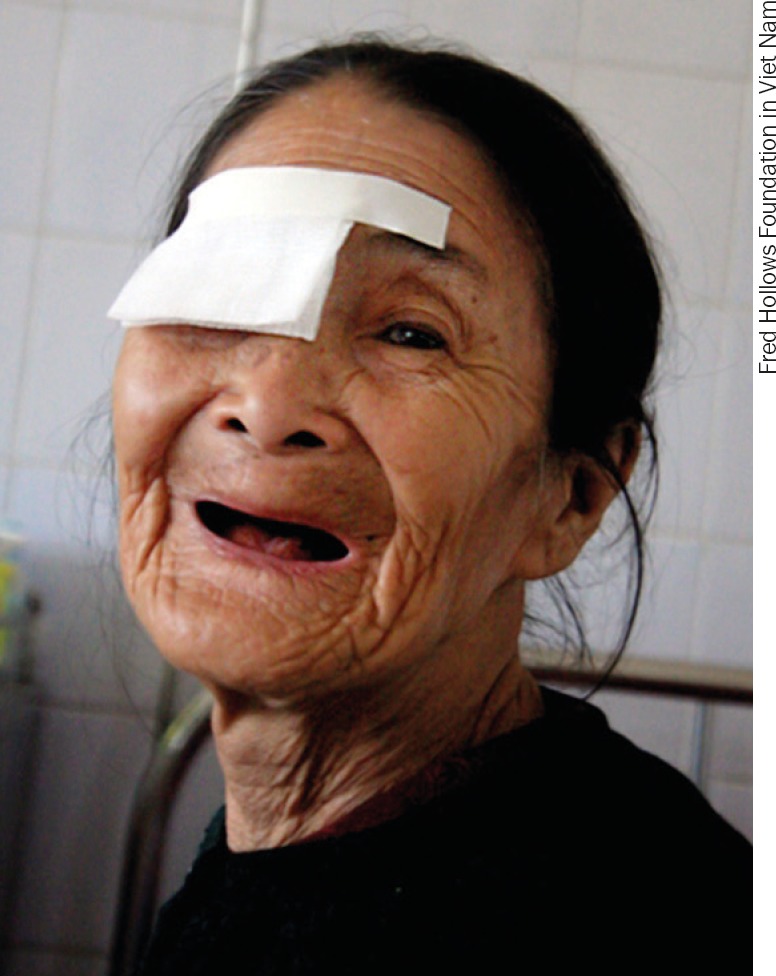
A patient after cataract surgery

These initiatives form part of the preparations for handover of the project to local surgeons once FHF support comes to an end.

## Sustainable income sources

Viet Nam has a government health insurance plan with a co-payment scheme which covers 70–80% of the costs of cataract operations and eye disease treatments.

According to a report by Quang Nam Social Insurances in 2009, 67% of the population had health insurance cards. Health insurance enables more patients to go to eye units for screening and treatment, and higher patient numbers boosts hospital income.

The CEC project also has encouraged local authorities to contribute matched funding for eye facility construction. In Dai Loc, the local government contributed 20% of the construction cost of the eye unit, while in Que Son the local government paid the full cost of construction (US $100,000).

Sales of spectacles are an additional source of income. The district hospitals provide high quality refraction services for post-operative patients at a cost of just US $6–10 per pair. This also helps to ensure that patients get the best visual outcomes.

With its strategy for sustainability, the CEC project in Que Son and Dai Loc has developed high quality, affordable services and helps to narrow the gap between eye care services and patients.

